# Supplementary material for: Cell-Type-Specific Gene Regulatory Networks of Pro-Inflammatory and Pro-Resolving Lipid Mediator Biosynthesis in the Immune System
Source: Int J Mol Sci. 2023 Feb 22;24(5):4342. doi: 10.3390/ijms24054342 (PMC10001763; doi:10.3390/ijms24054342)
Supplement: Supplementary file 1 [file ijms-24-04342-s001.zip › Hoch_et_al_LipidNetworks_Supplementary File S1.pdf]

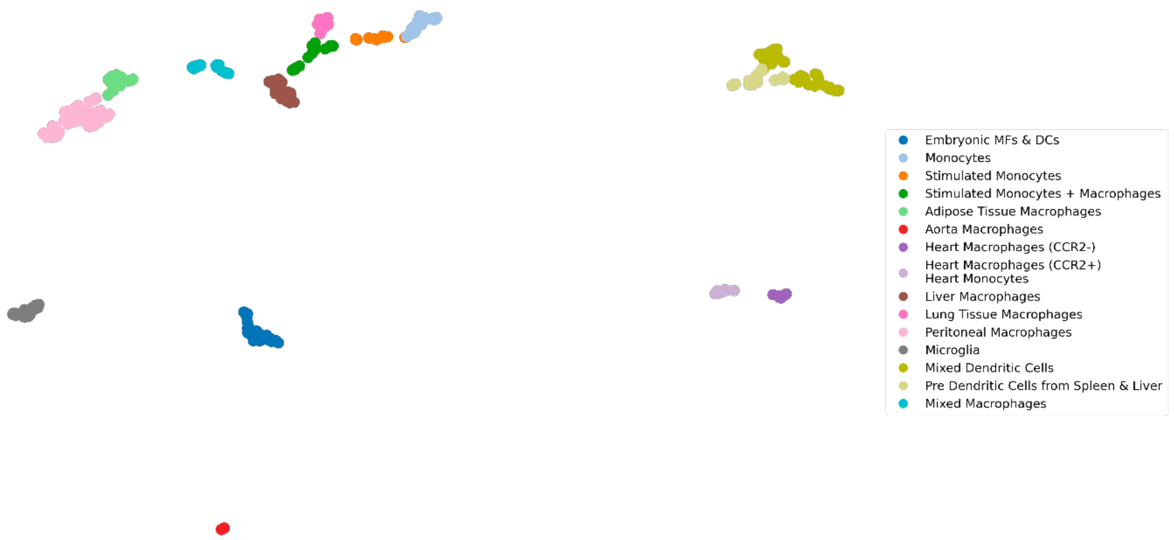

Cluster Cell Type Majority	Top 10 Genes	Sample Labels (as defined in the GEO)
**Embryonic MFs & DCs**	MEN1<br>E2F8<br>DMAP1<br>TRIM28<br>FOXM1<br>HIF3A<br>TFDP1<br>WDR5<br>HCFC1<br>MYB	MF.E10.5.YS.1<br>MF.E10.5.YS.2<br>DC.pDC.DN.BM.1<br>DC.pDC.DN.BM.2<br>DC.pDC.DP.BM.2<br>DC.pDC.DP.BM.3<br>DC.pDC.SigHp6Cn.BM.2<br>DC.pDC.SigHp6Cn.BM.3<br>DC.pDC.SigHn6Cp.BM.1<br>DC.pDC.SigHn6Cp.BM.2<br>DC.pDC.SigHn6Cp.BM.3<br>MF.64p6Cn6Gn.E14.5.Kd.1<br>MF.64p6Cn6Gn.E14.5.Kd.2<br>MF.64p6Cn6Gn.E14.5.Kd.3<br>MF.64p6Cn6Gn.E14.5.Lu.1<br>MF.64p6Cn6Gn.E14.5.Lu.2<br>MF.64p6Cn6Gn.E14.5.Lu.3<br>MF.64p6Cn6Gn.E16.5.Kd.1<br>MF.64p6Cn6Gn.E16.5.Kd.2<br>MF.64p6Cn6Gn.E16.5.Lu.1<br>MF.64p6Cn6Gn.E16.5.Lu.2<br>MF.64p6Cn6Gn.E18.5.Br.1<br>MF.64p6Cn6Gn.E18.5.Br.2<br>MF.64p6Cn6Gn.E18.5.Co.2<br>MF.64p6Cn6Gn.E18.5.Co.3<br>MF.64p6Cn6Gn.E18.5.Dr.1<br>MF.64p6Cn6Gn.E18.5.Dr.2<br>MF.64p6Cn6Gn.E18.5.Ep.1<br>MF.64p6Cn6Gn.E18.5.Ep.2<br>MF.64p6Cn6Gn.E18.5.F.Ht.1<br>MF.64p6Cn6Gn.E18.5.F.Ht.2<br>MF.64p6Cn6Gn.E18.5.Kd.1<br>MF.64p6Cn6Gn.E18.5.Kd.2<br>MF.64p6Cn6Gn.E18.5.Sp.1<br>MF.64p6Cn6Gn.E18.5.Sp.2<br>MF.64p6Cn6Gn.E18.5.Thy.1<br>MF.64p6Cn6Gn.E18.5.Thy.2<br>MF.64p6Cn6Gn.E18.5.Thy.3<br>MF.64p6Cn6Gn.Kd.Neo.1<br>MF.64p6Cn6Gn.Kd.Neo.2<br>MF.KC.Clec4FpTim4p64p.APAP.36h.Lv.1<br>MF.KC.Clec4FpTim4p64p.APAP.36h.Lv.3<br>MF.KC.Clec4FpTim4p64p.APAP.36h.Lv.4<br>MF.45lo.Calb.48h.CNS.1

**Monocytes**	NUPR1<br>KLF2<br>POU2F2<br>STAT5B	Mo.6Cp.Bl.6<br>Mo.6Cp.Bl.7<br>Mo.6Cp.Bl.8<br>Mo.6Cp.Thio.24h.Bl.1<br>Mo.6Cp.Thio.24h.Bl.2<br>Mo.6Cp.Thio.24h.Bl.3<br>Mo.6Cp.Thio.72h.Bl.1<br>Mo.6Cp.Thio.72h.Bl.2<br>Mo.6Cp.Thio.72h.Bl.3<br>Mo.6CpThio.8h.Bl.1<br>Mo.6CpThio.8h.Bl.2<br>Mo.6CpThio.8h.Bl.3<br>Mo.6Chi11bp.PBS.Lv.2<br>Mo.6Chi11bp.PBS.Lv.3<br>Mo.6Chi11bp.PBS.Lv.4<br>Mo.6Cn.Bl.3<br>Mo.6Cn.Lu.1<br>Mo.6Cn.Lu.2<br>Mo.6Cn.Lu.3<br>Mo.6Cp.Bl.3<br>Mo.6Cp.Bl.4<br>Mo.6Cp.Bl.5<br>Mo.6Cp.Lu.1<br>Mo.6Cp.Lu.2<br>Mo.6Cp.Lu.3<br>Mo.6Cn.Bl.1<br>Mo.6Cn.Bl.2<br>Mo.6Cp.Bl.1<br>Mo.6Cp.Bl.2
**Stimulated Monocytes**	LIMD1<br>PHB2<br>ARNT<br>NFKB2<br>ESRRA<br>SMAD2<br>NFYA<br>AHR<br>CEBPB<br>HIF1A	Mo.115pICAM2n226n6Cn.Thio.4h.PC.1<br>Mo.115pICAM2n226n6Cn.Thio.4h.PC.2<br>Mo.115pICAM2n226n6Cn.Thio.4h.PC.3<br>Mo.115pICAM2n226n6Cn.Thio.8h.PC.1<br>Mo.115pICAM2n226n6Cn.Thio.8h.PC.2<br>Mo.115pICAM2n226n6Cn.Thio.8h.PC.3<br>Mo.6CpThio.4h.Bl.2<br>Mo.6CpThio.4h.Bl.3<br>Mo.6Chi11bp.APAP.12h.Lv.1<br>Mo.6Chi11bp.APAP.12h.Lv.2<br>Mo.6Chi11bp.APAP.12h.Lv.3<br>Mo.6Chi11bp.APAP.12h.Lv.4<br>Mo.6Chi11bp.PBS.Lv.1<br>Mo.6Cp.Lu.4<br>Mo.6Cp.Lu.5
**Stimulated Monocytes + Macrophages**	IRF3<br>CREB5<br>REST<br>TRIB3<br>IRF7	MF.64p.Th.1<br>MF.64p.Th.2<br>MF.64p.Th.3<br>MF.64p.Th.4<br>Mo.6Chi11bp.APAP.36h.Lv.1<br>Mo.6Chi11bp.APAP.36h.Lv.2<br>Mo.6Chi11bp.APAP.36h.Lv.3<br>Mo.6Chi11bp.APAP.36h.Lv.4<br>MF.64p6Cn206nIIp.LPS.d3.Lu.2<br>MF.SSChipSigFn.LPS.d3.BAL.1<br>MF.SSChipSigFn.LPS.d3.BAL.2<br>MF.SSChipSigFn.LPS.d6.BAL.1<br>MF.SSChipSigFn.LPS.d6.BAL.2<br>Mo.64p6CpIIp.LPS.d3.Lu.1<br>Mo.64p6CpIIp.LPS.d3.Lu.2<br>Mo.64p6CpIIp.LPS.d6.Lu.1<br>Mo.SSClop6Cp.LPS.d3.BAL.1<br>Mo.SSClop6Cp.LPS.d3.BAL.2<br>MF.pIC.alv.siglecFp.Lu.2
**Adipose Tissue Macrophages**	MEN1<br>ZBTB16	MF.226pIIp.PC.1<br>MF.226pIIp.PC.3<br>MF.226pIIp.PC.4

	CEBPA<br>RUNX1<br>CEBPD	MF.226pIIp480lo.PC.1<br>MF.226pIIp480lo.PC.2<br>MF.480p64pMerTKp.WAT.1<br>MF.480p64pMerTKp.WAT.2<br>MF.480p64pMerTKp.WAT.3<br>MF.480p64pLyve1p.MS.1<br>MF.480p64pLyve1p.MS.2<br>MF.480p64pLyve1p.MS.3<br>MF.480p64pLyve1p.MS.4<br>MF.480p64pMerTKp.AT.1<br>MF.480p64pMerTKp.AT.2<br>MF.480p64pMerTKp.AT.3<br>MF.480p64pMerTKp.MAT.1<br>MF.480p64pMerTKp.MAT.2<br>MF.480p64pMerTKp.MAT.3<br>MF.480p64pMerTKp.MAT.4<br>MF.alv.11cp64pSiglecFp.Lu.1<br>MF.alv.11cp64pSiglecFp.Lu.2<br>MF.alv.11cp64pSiglecFp.Lu.3<br>MF.DRG.1<br>MF.DRG.4<br>MF.SN.3
**Aorta Macrophages**	ZBTB7A<br>TRRAP<br>MEF2C<br>SMAD4<br>NCOR1<br>FOXO1<br>DRAP1<br>ATF1<br>ESR1<br>MAML1	MF.64pLYVEpIIn.Ao.1<br>MF.64pLYVEpIIn.Ao.2<br>MF.64pLYVEpIIn.Ao.3<br>MF.64pLYVEpIIp.Ao.1<br>MF.64pLYVEpIIp.Ao.2<br>MF.64pLYVEpIIp.Ao.3<br>MF.64pLYVEnIIp.Ao.1<br>MF.64pLYVEnIIp.Ao.2<br>MF.64pLYVEnIIp.Ao.3
**Heart Macrophages (CCR2-)**	MAML1<br>FOXO1<br>FLI1<br>CLOCK<br>GTF2A1<br>JDP2<br>FOXO3<br>NFATC2<br>TOP2B<br>HDAC7	MF.CCR2p64p6ClonGFPp.Ht.2<br>MF.CCR2nFlt3n64p6ClonGFPntdTomaton.Ht.1<br>MF.CCR2nFlt3n64p6ClonGFPntdTomaton.Ht.2<br>MF.CCR2nFlt3n64p6ClonGFPntdTomaton.Ht.3<br>MF.CCR2nFlt3n64p6ClonGFPntdTomaton.Ht.4<br>MF.CCR2nFlt3n64p6ClonGFPntdTomaton.inj.d2.Ht.1<br>MF.CCR2nFlt3n64p6ClonGFPntdTomaton.inj.d2.Ht.2<br>MF.CCR2nFlt3n64p6ClonGFPntdTomaton.inj.d2.Ht.3<br>MF.CCR2nFlt3n64p6ClonGFPntdTomaton.inj.d2.Ht.4<br>MF.CCR2nFlt3n64p6ClonGFPntdTomatop.Ht.1<br>MF.CCR2nFlt3n64p6ClonGFPntdTomatop.Ht.2
**Heart Macrophages (CCR2+) & Heart Monocytes**	HDAC5<br>ATF6<br>XBP1<br>HIPK2<br>TFPT<br>HSF2<br>JUND<br>PIAS4<br>BACH2<br>NFKB2	MF.CCR2p64p6ClonGFPp.Ht.3<br>MF.CCR2p64p6ClonGFPp.inj.d2.Ht.1<br>MF.CCR2p64p6ClonGFPp.inj.d2.Ht.2<br>MF.CCR2p64p6ClonGFPp.inj.d2.Ht.3<br>MF.CCR2p64p6ClonGFPp.inj.d2.Ht.4<br>MF.CCR2p64p6ClonGFPp.inj.d4.Ht.1<br>MF.CCR2p64p6ClonGFPp.inj.d4.Ht.2<br>MF.CCR2p64p6ClonGFPp.inj.d4.Ht.3<br>Mo.64p6ChinGFPp.inj.d2.Ht.1<br>Mo.64p6ChinGFPp.inj.d2.Ht.2<br>Mo.64p6ChinGFPp.inj.d2.Ht.3<br>Mo.64p6ChinGFPp.inj.d2.Ht.4<br>Mo.64p6ChinGFPp.inj.d4.Ht.1<br>Mo.64p6ChinGFPp.inj.d4.Ht.2<br>Mo.64p6ChinGFPp.inj.d4.Ht.3<br>Mo.64p6ChinGFPp.inj.d4.Ht.4

**Liver Macrophages**	SP100<br>GATA3	MF.64p6Cn6Gn.E18.5.Lv.1<br>MF.64p6Cn6Gn.E18.5.Lv.2<br>MF.64p6Cn6Gn.Lu.Neo.2<br>MF.KC.Clec4FpTim4p64p.APAP.12h.Lv.1<br>MF.KC.Clec4FpTim4p64p.APAP.12h.Lv.2<br>MF.KC.Clec4FpTim4p64p.APAP.12h.Lv.4<br>MF.KC.Clec4FpTim4p64p.APAP.36h.Lv.2<br>MF.KC.Clec4FpTim4p64p.Lv.2<br>MF.KC.Clec4FpTim4p64p.Lv.3<br>MF.KC.Clec4FpTim4p64p.Lv.4<br>MF.KC.Clec4FpTim4p64p.PBS.Lv.1<br>MF.KC.Clec4FpTim4p64p.PBS.Lv.2<br>MF.KC.Clec4FpTim4p64p.PBS.Lv.3<br>MF.KC.Clec4FpTim4p64p.PBS.Lv.4<br>Mo.6Cint11cint.APAP.36h.Lv.1<br>Mo.6Cint11cint.APAP.36h.Lv.2<br>Mo.6Cint11cint.APAP.36h.Lv.3<br>Mo.6Cint11cint.APAP.36h.Lv.4<br>MF.11blop64p169p.Calb.48h.Lv.1<br>MF.11blop64p169p.Calb.48h.Lv.2<br>MF.11blop64p169p.Calb.8h.Lv.1<br>MF.11blop64p169p.Calb.8h.Lv.2<br>MF.11blop64p169p.Lv.1<br>MF.11blop64p169p.Lv.2<br>MF.6Gn480hi.Calb.48h.Kd.1<br>MF.480p.SP.1<br>MF.480p.SP.2<br>MF.64pMerTKp11blo.Sp.1<br>MF.64pMerTKp11blo.Sp.2
**Lung Tissue Macrophages**	n.s.	MF.64p6Cn206pIIn.LPS.d3.Lu.1<br>MF.64p6Cn206pIIn.LPS.d3.Lu.2<br>MF.64p6Cn206pIIn.LPS.d6.Lu.1<br>MF.64p6Cn206pIIn.LPS.d6.Lu.2<br>MF.64p6Cn206pIIn.Lu.1<br>MF.64p6Cn206pIIn.Lu.2<br>MF.64p6Cn206pIIp.LPS.d3.Lu.1<br>MF.64p6Cn206pIIp.LPS.d3.Lu.2<br>MF.64p6Cn206pIIp.LPS.d6.Lu.1<br>MF.64p6Cn206pIIp.LPS.d6.Lu.2<br>MF.64p6Cn206nIIp.LPS.d3.Lu.1<br>MF.64p6Cn206nIIp.LPS.d6.Lu.1<br>MF.64p6Cn206nIIp.LPS.d6.Lu.2<br>MF.64p6Cn206nIIp.Lu.1<br>MF.64p6Cn206nIIp.Lu.2<br>Mo.64p6CpIIp.LPS.d6.Lu.2<br>MF.6Gn480hi.Calb.8h.Kd.1<br>MF.6Gn480hi.Calb.8h.Kd.2<br>MF.6Gn480hi.Kd.1<br>MF.6Gn480hi.Kd.2<br>MF.SN.1
**Peritoneal Macrophages**	n.s.	MF.PC.44<br>MF.PC.45<br>MF.PC.46<br>MF.F.PC.1<br>MF.PC.47<br>MF.PC.48<br>MF.PC.49<br>MF.PC.50<br>MF.PC.21<br>MF.PC.09<br>MF.PC.10<br>MF.PC.03<br>MF.PC.04<br>MF.PC.05<br>MF.PC.06<br>MF.115pICAM2p226n6Cn.PC.1<br>MF.115pICAM2p226n6Cn.PC.2<br>MF.115pICAM2p226n6Cn.PC.3

		MF.64p6Cn6Gn.E18.5.PC.1<br>MF.64p6Cn6Gn.E18.5.PC.2<br>MF.E18.5.F.PC.1<br>MF.E18.5.F.PC.2<br>MF.PC.37<br>MF.PC.38<br>MF.PC.17<br>MF.PC.07<br>MF.PC.08<br>MF.PC.19<br>MF.PC.20<br>MF.PC.02<br>MF.PC.23<br>MF.PC.24<br>MF.PC.39<br>MF.PC.40<br>MF.PC.25<br>MF.PC.26<br>MF.B220n6Cn64pTim4p.PC.1<br>MF.B220n6Cn64pTim4p.PC.2<br>MF.B220n6Cn64pTim4p.PC.3<br>MF.B220n6Cn64pTim4p.PC.4<br>MF.B220n6Cn64pTim4p.PC.5<br>MF.B220n6Cn64pTim4p.PC.6<br>MF.PC.11<br>MF.PC.12<br>MF.PC.13<br>MF.PC.14<br>MF.PC.15<br>MF.F.PC.2<br>MF.F.PC.3<br>MF.PC.51<br>MF.PC.52<br>MF.E14.5.F.PC.1<br>MF.E14.5.F.PC.2<br>MF.E14.5.F.PC.3<br>MF.E16.5.PC.1<br>MF.E16.5.PC.2<br>MF.E18.5.F.PC.3<br>MF.PC.F.Neo.1<br>MF.PC.F.Neo.2<br>MF.PC.18<br>MF.45lo.Calb.48h.CNS.2<br>MF.PC.01<br>MF.B220n6Cn64pTim4n.PC.1<br>MF.B220n6Cn64pTim4n.PC.2<br>MF.B220n6Cn64pTim4n.PC.3
Microglia	XRCC5<br>MEF2A<br>SMAD3<br>HDAC11<br>MEF2C<br>ZNF24<br>PRDM1<br>ZFHX3<br>ARID1A<br>ETS1	MF.45lo.Calb.8h.CNS.1<br>MF.45lo.Calb.8h.CNS.2<br>MF.45lo.CNS.1<br>MF.45lo.CNS.2<br>MF.microglia.cerebel.CNS.1<br>MF.microglia.cerebel.CNS.2<br>MF.microglia.cerebr.CNS.1<br>MF.microglia.cerebr.CNS.2<br>MF.microglia.cerebr.CNS.3<br>MG.cerebel.1<br>MG.cerebel.2<br>MG.cerebel.3<br>MG.cortex.1<br>MG.cortex.2<br>MG.cortex.3<br>MG.hippo.1<br>MG.hippo.2<br>MG.hippo.3<br>MF.microglia.CNS.1<br>MF.microglia.CNS.2<br>MF.microglia.CNS.3<br>MF.microglia.CNS.4<br>MF.microglia.CNS.5

		MG.SC.1<br>MG.SC.2<br>MG.SC.3
Mixed Dendritic Cells	ZEB1<br>PIAS3<br>CREB1<br>CIITA<br>ETV3<br>IRF2<br>NCOA3<br>RFX5	DC.24p8apXCR1n.Sp.1<br>DC.24p8apXCR1n.Sp.2<br>DC.24p8apXCR1p.Sp.1<br>DC.24p8apXCR1p.Sp.2<br>DC.24p8anXCR1n.Sp.1<br>DC.24p8anXCR1n.Sp.2<br>DC.24p8anXCR1p.Sp.1<br>DC.24p8anXCR1p.Sp.2<br>DC.XCR1pSIRPan.Th.1<br>DC.XCR1pSIRPan.Th.2<br>DC.XCR1pSIRPan.Th.3<br>DC.XCR1pSIRPan.Th.4<br>DC.cDC1.XCR1p.Lv.1<br>DC.cDC1.XCR1p.Lv.2<br>DC.cDC1.XCR1p.Lv.3<br>DC.103p11bnsiglecFn.Lu.4<br>DC.103p11bnsiglecFn.Lu.5<br>DC.103p11bnsiglecFn.Lu.6<br>DC.8p4n.Sp.1<br>DC.8p4n.Sp.2<br>DC.8p4n.Sp.3<br>DC.res.11bp.LuLN.3<br>DC.8ap.MLN.1<br>DC.8ap.MLN.2<br>DC.8ap.PP.1<br>DC.8an103n.PP.1<br>DC.8an103n11bn.MLN.1<br>DC.8an103n11bn.MLN.2<br>DC.SigHn.64n11chipBst2n11bp8anRFPn.Sp.2<br>DC.SigHn.64n11chipBst2n11bp8anRFPn.Sp.3<br>DC.SigHn.64n11chipBst2n11bp8anRFPp.Sp.2<br>DC.SigHn.64n11chipBst2n11bp8anRFPp.Sp.3<br>DC.XCR1nSIRPap.Th.1<br>DC.XCR1nSIRPap.Th.2<br>DC.XCR1nSIRPap.Th.3<br>DC.XCR1nSIRPap.Th.4<br>DC.cDC2.172ap.Lv.1<br>DC.cDC2.172ap.Lv.2<br>DC.cDC2.172ap.Lv.3<br>DC.cDC2.172ap.Lv.4<br>DC.103n11bpsiglecFn.Lu.4<br>DC.103n11bpsiglecFn.Lu.5<br>DC.103n11bpsiglecFn.Lu.6<br>DC.mig.103p.LuLN.1<br>DC.mig.103p.LuLN.2<br>DC.mig.103p.LuLN.3<br>DC.mig.11bp.LuLN.1<br>DC.mig.11bp.LuLN.2<br>DC.8an103n.MLN.1<br>DC.8an103n.MLN.2<br>DC.8an103n.MLN.3<br>DC.8an103p.MLN.1<br>DC.8an103p.MLN.2<br>DC.8an103p.MLN.3<br>DC.8an103p.MLN.4
Pre Dendritic Cells from Spleen & Liver	SPIB<br>EHMT2<br>HIC1<br>PIR	DC.pDC.DN.Sp.1<br>DC.pDC.DN.Sp.2<br>DC.pDC.DN.Sp.3<br>DC.pDC.SigHn6Cp.Sp.1<br>DC.pDC.SigHn6Cp.Sp.2<br>DC.pDC.SigHn6Cp.Sp.3<br>DC.pDC.120g8p11cintp6Cp.Lv.1<br>DC.pDC.120g8p11cintp6Cp.Lv.2<br>DC.pDC.120g8p11cintp6Cp.Lv.3<br>DC.pDC.120g8p11cintp6Cp.Lv.4

		DC.res.11bp.LuLN.1<br>DC.res.11bp.LuLN.2<br>DC.res.8p.LuLN.1<br>DC.res.8p.LuLN.2<br>DC.res.8p.LuLN.3<br>DC.8ap.MLN.3<br>DC.8ap.MLN.4<br>DC.8ap.PP.3<br>DC.8ap.PP.4<br>DC.8an103n.PP.3<br>DC.8an103n.PP.4<br>DC.8an103p.PP.1<br>DC.8an103p.PP.3<br>DC.8an103p.PP.4<br>DC.8an103n11bn.PP.1<br>DC.8an103n11bn.PP.3<br>DC.8an103n11bn.PP.4
Mixed Macrophages	n.s.	MF.d6.EB.1<br>MF.d6.EB.2<br>MF.d8.EB.1<br>MF.d8.EB.2<br>MF.115p36p6ClopICAM2n226n.Thio.72h.PC.1<br>MF.115p36p6ClopICAM2n226n.Thio.72h.PC.2<br>MF.115p36p6ClopICAM2n226n.Thio.72h.PC.3<br>MF.115p36p6ClopICAM2n226n.Thio.24h.PC.1<br>MF.115p36p6ClopICAM2n226n.Thio.24h.PC.2<br>MF.115p36p6ClopICAM2n226n.Thio.24h.PC.3<br>Mo.115p6Cp36lopICAM2n226n.Thio.24h.PC.1<br>Mo.115p6Cp36lopICAM2n226n.Thio.24h.PC.2<br>Mo.115p6Cp36lopICAM2n226n.Thio.24h.PC.3<br>MF.64p6Cn6Gn.Lu.Neo.1<br>MF.11cpSigFp.BAL.1<br>MF.11cpSigFp.BAL.2<br>MF.11cpSigFp.LPS.d6.BAL.1<br>MF.11cpSigFp.LPS.d6.BAL.2<br>MF.6Gn480hi.Calb.48h.Kd.2<br>MF.alv.6GnSigFp11cp.Lu.1<br>MF.alv.6GnSigFp11cp.Lu.2
Embryonic MFs & DCs	MEN1<br>E2F8<br>DMAP1<br>TRIM28<br>FOXM1<br>HIF3A<br>TFDP1<br>WDR5<br>HCFC1<br>MYB	MF.E10.5.YS.1<br>MF.E10.5.YS.2<br>DC.pDC.DN.BM.1<br>DC.pDC.DN.BM.2<br>DC.pDC.DP.BM.2<br>DC.pDC.DP.BM.3<br>DC.pDC.SigHp6Cn.BM.2<br>DC.pDC.SigHp6Cn.BM.3<br>DC.pDC.SigHn6Cp.BM.1<br>DC.pDC.SigHn6Cp.BM.2<br>DC.pDC.SigHn6Cp.BM.3<br>MF.64p6Cn6Gn.E14.5.Kd.1<br>MF.64p6Cn6Gn.E14.5.Kd.2<br>MF.64p6Cn6Gn.E14.5.Kd.3<br>MF.64p6Cn6Gn.E14.5.Lu.1<br>MF.64p6Cn6Gn.E14.5.Lu.2<br>MF.64p6Cn6Gn.E14.5.Lu.3<br>MF.64p6Cn6Gn.E16.5.Kd.1<br>MF.64p6Cn6Gn.E16.5.Kd.2<br>MF.64p6Cn6Gn.E16.5.Lu.1<br>MF.64p6Cn6Gn.E16.5.Lu.2<br>MF.64p6Cn6Gn.E18.5.Br.1<br>MF.64p6Cn6Gn.E18.5.Br.2<br>MF.64p6Cn6Gn.E18.5.Co.2<br>MF.64p6Cn6Gn.E18.5.Co.3<br>MF.64p6Cn6Gn.E18.5.Dr.1<br>MF.64p6Cn6Gn.E18.5.Dr.2<br>MF.64p6Cn6Gn.E18.5.Ep.1<br>MF.64p6Cn6Gn.E18.5.Ep.2<br>MF.64p6Cn6Gn.E18.5.F.Ht.1

		MF.64p6Cn6Gn.E18.5.F.Ht.2<br>MF.64p6Cn6Gn.E18.5.Kd.1<br>MF.64p6Cn6Gn.E18.5.Kd.2<br>MF.64p6Cn6Gn.E18.5.Sp.1<br>MF.64p6Cn6Gn.E18.5.Sp.2<br>MF.64p6Cn6Gn.E18.5.Thy.1<br>MF.64p6Cn6Gn.E18.5.Thy.2<br>MF.64p6Cn6Gn.E18.5.Thy.3<br>MF.64p6Cn6Gn.Kd.Neo.1<br>MF.64p6Cn6Gn.Kd.Neo.2<br>MF.KC.Clec4FpTim4p64p.APAP.36h.Lv.1<br>MF.KC.Clec4FpTim4p64p.APAP.36h.Lv.3<br>MF.KC.Clec4FpTim4p64p.APAP.36h.Lv.4<br>MF.45lo.Calb.48h.CNS.1
