# Supplementary material for: Cell-Type-Specific Gene Regulatory Networks of Pro-Inflammatory and Pro-Resolving Lipid Mediator Biosynthesis in the Immune System
Source: Int J Mol Sci. 2023 Feb 22;24(5):4342. doi: 10.3390/ijms24054342 (PMC10001763; doi:10.3390/ijms24054342)
Supplement: Supplementary file 1 [file ijms-24-04342-s001.zip › Hoch_et_al_LipidNetworks_Supplementary File S2.pdf]

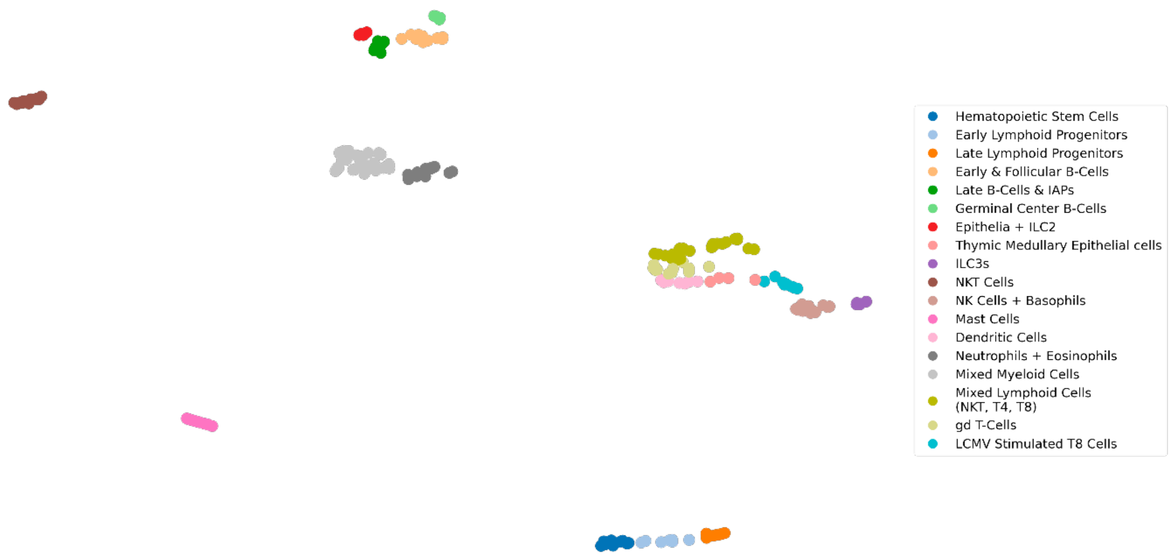

**Cluster Cell Type Majority**	**Top 10 Genes**	**Sample Labels (as defined in the GEO)**
**Hematopoietic Stem Cells**	SUZ12<br>HHEX<br>RXRA<br>GFI1B<br>NCOR2<br>POU2F1<br>BCL11A<br>SPEN<br>NFYB<br>KAT6A	LTHSC.34-.BM.1<br>LTHSC.34-.BM.2<br>LTHSC.34+.BM.1<br>LTHSC.34+.BM.2<br>MMP2.150+48+.BM.1<br>MMP3.48+.BM.1<br>MMP3.48+.BM.2<br>MMP4.135+.BM.1<br>MMP4.135+.BM.2<br>STHSC.150-.BM.1<br>STHSC.150-.BM.2
**Early Lymphoid Progenitors**	BARD1<br>RUNX2<br>TRPS1<br>HMGB2<br>BIN1<br>MYCN<br>FOXM1<br>BRIP1<br>MEF2C<br>MECP2	preT.DN1.Th.1<br>preT.DN1.Th.2<br>preT.DN2a.Th.2<br>proB.CLP.BM.1<br>proB.CLP.BM.2<br>proB.FrA.BM.1<br>proB.FrA.BM.2
**Late Lymphoid Progenitors**	HMG20B<br>BRCA2<br>HCFC1<br>TFDP1<br>UHRF1<br>DMAP1<br>POLR1A<br>MEN1	preT.DN2b.Th.1<br>preT.DN2b.Th.2<br>preT.DN3.Th.1<br>preT.DN3.Th.2<br>proB.FrBC.BM.1<br>proB.FrBC.BM.2<br>T.DN4.Th.1<br>T.DN4.Th.2<br>T.ISP.Th.1<br>T.ISP.Th.2

	EAF1<br>ELK1	
**Early & Follicular B-Cells**	POU2AF1<br>LMO2<br>IRF4<br>HDAC9<br>PML	B.Fem.Sp.1<br>B.Fo.Sp.1<br>B.Fo.Sp.2<br>B.Fo.Sp.3<br>B.Fo.Sp.4<br>B.FrE.BM.1<br>B.FrE.BM.2<br>B.mem.Sp.1<br>B.Sp.4<br>B.T1.Sp.2<br>B.T2.Sp.1<br>B.T2.Sp.2<br>B.T3.Sp.2
**Late B-Cells & IAPs**	BACH2<br>PAX5<br>MBD1	B.MZ.Sp.1<br>B.MZ.Sp.2<br>B.PC.BM.1<br>B1b.PC.1<br>B1b.PC.2<br>DC.pDC.Sp.1<br>DC.pDC.Sp.2<br>IAP.SLN.1<br>IAP.SLN.2
**Germinal Center B-Cells**	TP53BP1<br>CTCF<br>E2F6<br>CTBP1<br>BRCA1<br>FOXK2<br>NCOA3<br>DMAP1<br>SIN3A<br>CEBPZ	B.GC.CB.Sp.1<br>B.GC.CB.Sp.2<br>B.GC.CB.Sp.3<br>B.GC.CC.Sp.1<br>B.GC.CC.Sp.2<br>B.GC.CC.Sp.3
**Epithelia + ILC2**	IRF2<br>IRF8<br>IRF1<br>CREB1<br>IRF4<br>USF1<br>ABL1	DC.4+.Sp.1<br>DC.4+.Sp.2<br>DC.4+.Sp.3<br>DC.8+.Sp.1<br>DC.8+.Sp.2<br>DC.8+.Sp.3
**Thymic Medullary Epithelial cells**	ZNF423<br>PIAS4<br>PER2<br>GTF2I<br>ING1<br>HOPX<br>SPIB<br>SNAI2<br>PIR	Ep.MECHi.Th.1<br>Ep.MECHi.Th.2<br>LEC.SLN.3<br>Treg.4.FP3+.Nrplo.Co.2

**ILC3s**	EHMT2<br>PARP1<br>ZBTB14<br>TOP2B<br>SRF<br>NFIC<br>MEF2A<br>NFRKB<br>KLF4<br>JUN	ILC3.CCR6+.SI.1<br>ILC3.CCR6+.SI.2<br>ILC3.NKp46+.SI.1<br>ILC3.NKp46+.SI.2<br>ILC3.NKp46-CCR6-.SI.2
**NKT Cells**	RFX1<br>HSF4<br>ETS1<br>RARA<br>RELA	NKT.19-8-TCRb+CD1daGalCerTet+.Lu.1<br>NKT.19-8-TCRb+CD1daGalCerTet+.Lu.2<br>NKT.19-8-TCRb+CD1daGalCerTet+.Sp.1<br>NKT.19-8-TCRb+CD1daGalCerTet+.Sp.2<br>NKT.19-8-TCRb+CD1daGalCerTet+.Sp.3<br>NKT.19-8-TCRb+CD1daGalCerTet+.Th.1<br>NKT.19-8-TCRb+CD1daGalCerTet+.Th.2<br>NKT.19-8-TCRb+CD1daGalCerTet+.Th.3<br>NKT.19-8-TCRb+CD1daGalCerTet+.Lv.1<br>NKT.19-8-TCRb+CD1daGalCerTet+.Lv.2<br>NKT.19-8-TCRb+CD1daGalCerTet+.Lv.3<br>T.4.19-8-TCRb+CD4+.Sp.1<br>T.4.19-8-TCRb+CD4+.Sp.2
**NK Cells + Basophils**	SKI<br>GATA1	NK.27+11b-.BM.1<br>NK.27+11b-.BM.2<br>NK.27+11b-.Sp.1<br>NK.27+11b-.Sp.2<br>NK.27+11b+.BM.1<br>NK.27+11b+.BM.2<br>NK.27+11b+.Sp.1<br>NK.27+11b+.Sp.2<br>NK.27-11b+.BM.1<br>NK.27-11b+.BM.2<br>NK.27-11b+.Sp.2<br>Ba.Sp.1<br>Ba.Sp.2<br>Ba.Sp.3
**Mast Cells**	n.s.	MC.heparinase.PC.3<br>MC.SCA1hi.CX3CR1hi.PC.1<br>MC.SCA1hi.CX3CR1hi.PC.2<br>MC.SCA1hi.CX3CR1hi.PC.3<br>MC.SCA1hi.CX3CR1lo.PC.1<br>MC.SCA1hi.CX3CR1lo.PC.2<br>MC.SCA1hi.CX3CR1lo.PC.3<br>MC.SCA1lo.CX3CR1hi.PC.1<br>MC.SCA1lo.CX3CR1hi.PC.2<br>MC.SCA1lo.CX3CR1hi.PC.3<br>MC.SCA1lo.CX3CR1lo.PC.1<br>MC.SCA1lo.CX3CR1lo.PC.2<br>MC.SCA1lo.CX3CR1lo.PC.3
**Dendritic Cells**	NCOA4<br>FOXO3<br>SMAD2<br>PA2G4<br>HNRNPD<br>RBPJ<br>IRF3<br>AHR	BEC.SLN.1<br>BEC.SLN.2<br>BEC.SLN.3<br>ILC2.SI.1<br>ILC2.SI.2<br>ILC2.ST2-.SI.2<br>ILC2.ST2-.SI.1<br>LEC.SLN.2

	KHDRBS1<br>CLOCK	
**Neutrophils + Eosinophils**	SP3<br>RB1CC1<br>RB1<br>NFATC1<br>SIRT3<br>MEN1<br>HDAC5<br>FOXO3<br>NF1<br>TRRAP	GN.BM.1<br>GN.BM.2<br>GN.Sp.3<br>GN.Sp.4<br>GN.Thio.PC.1<br>GN.Thio.PC.2<br>Eo.PC.1<br>Eo.PC.2<br>Eo.Sp.2<br>Eo.Sp.3<br>MC.Ht.1<br>MC.Ht.2
**Mixed Myeloid Cells**	POU2F2<br>CEBPD<br>HSF2<br>IRF7<br>HNRNPD<br>CREB5<br>HOPX	MF.Alv.Lu.1<br>MF.Alv.Lu.2<br>MF.Fem.PC.1<br>MF.Fem.PC.2<br>MF.PC.3<br>MF.PC.4<br>MF.pIC.Alv.Lu.2<br>MF.102+480+.PC.1<br>MF.102+480+.PC.2<br>MF.AT.1<br>MF.AT.2<br>MF.226+II+480lo.PC.1<br>MF.226+II+480lo.PC.2<br>Mo.6C-II-.Bl.1<br>Mo.6C-II-.Bl.2<br>Mo.6C+II-.Bl.1<br>Mo.6C+II-.Bl.2<br>B.PB.Sp.1<br>B.PB.Sp.2<br>B.PC.Sp.2<br>FRC.CD140a+.Madcam-.CD35-.SLN.1<br>FRC.CD140a+.Madcam-.CD35-.SLN.2<br>FRC.CD140a+.Madcam-.CD35-.SLN.3<br>MF.microglia.CNS.1<br>MF.microglia.CNS.2<br>MF.RP.Sp.1<br>MF.RP.Sp.2
**Mixed Lymphoid Cells (NKT<br>T4<br>T8)**	MCM5<br>HIF3A<br>PIAS1<br>HIF1A<br>PRDM1<br>NFKB2<br>IRF9<br>PTTG1<br>STAT2	NKT.Sp.LPS.18hr.1<br>NKT.Sp.LPS.18hr.2<br>NKT.Sp.LPS.3hr.1<br>NKT.Sp.LPS.3hr.2<br>T.4.Nve.Fem.Sp.1<br>T.4.Nve.Fem.Sp.2<br>T.4.Nve.Sp.1<br>T.4.Nve.Sp.2<br>T.8.Nve.Sp.1<br>T.8.Nve.Sp.2<br>T8.TN.P14.Sp.2<br>Treg.4.25hi.Sp.1<br>Treg.4.25hi.Sp.2<br>T.4.Sp.aCD3+CD40.18hr.1<br>T.4.Sp.aCD3+CD40.18hr.2<br>T.4.Th.1<br>T.4.Th.2<br>T.8.Th.1<br>T.8.Th.2<br>T.DP.Th.1<br>T.DP.Th.2<br>Tgd.g1.1+d1.24a+.Th.1<br>Tgd.g1.1+d1.24a+.Th.2

		Tgd.g2+d1.24a+.Th.1<br>Tgd.g2+d1.24a+.Th.2<br>Tgd.g2+d17.24a+.Th.2
**gd T-Cells**	n.s.	T8.Tcm.LCMV.d180.Sp.1<br>T8.Tcm.LCMV.d180.Sp.2<br>T8.Tem.LCMV.d180.Sp.2<br>T8.TN.P14.Sp.1<br>Tgd.g1.1+d1.LN.1<br>Tgd.g1.1+d1.LN.2<br>Tgd.g2+d1.LN.1<br>Tgd.g2+d1.LN.2<br>Tgd.g2+d17.LN.1<br>Tgd.g2+d17.LN.2<br>Tgd.Sp.3<br>Tgd.Sp.4
**LCMV Stimulated T8 Cells**	n.s.	NKT.Sp.3<br>NKT.Sp.LPS.3d.2<br>T8.IEL.LCMV.d7.Gut.1<br>T8.IEL.LCMV.d7.Gut.2<br>T8.MP.LCMV.d7.Sp.1<br>T8.MP.LCMV.d7.Sp.2<br>T8.TE.LCMV.d7.Sp.1<br>T8.TE.LCMV.d7.Sp.2<br>Treg.4.FP3+.Nrplo.Co.1
