# Supplementary figures and images for: Cell-Type-Specific Gene Regulatory Networks of Pro-Inflammatory and Pro-Resolving Lipid Mediator Biosynthesis in the Immune System
Source: Int J Mol Sci. 2023 Feb 22;24(5):4342. doi: 10.3390/ijms24054342 (PMC10001763; doi:10.3390/ijms24054342)

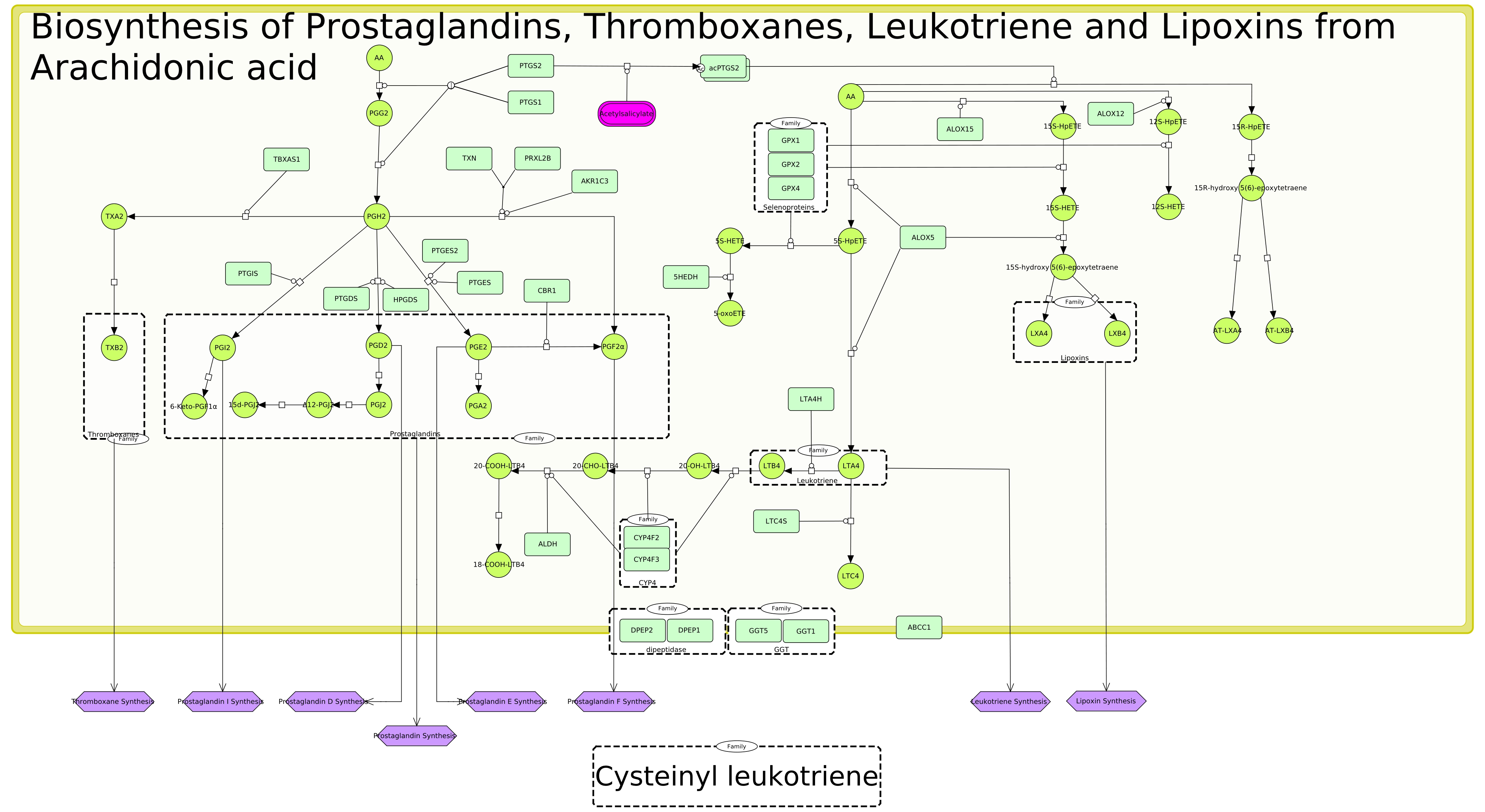

Supplement: Supplementary file 1 [file ijms-24-04342-s001.zip › FigureS1.jpg]

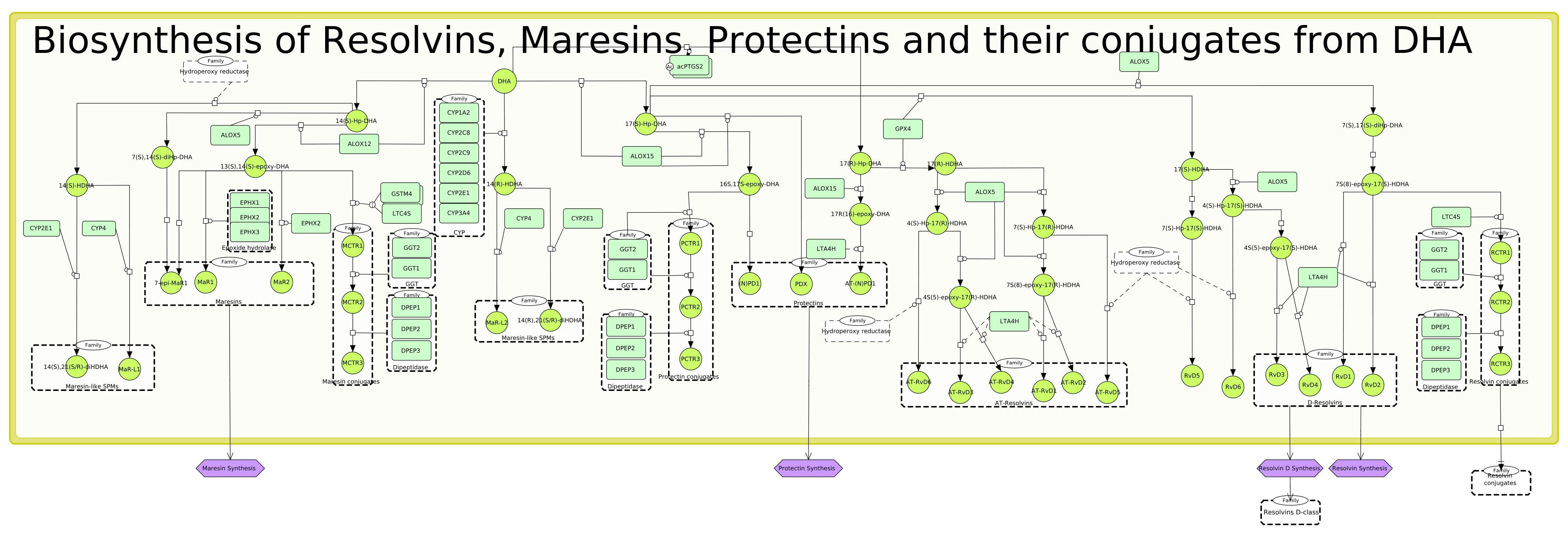

Supplement: Supplementary file 1 [file ijms-24-04342-s001.zip › FigureS2.jpg]

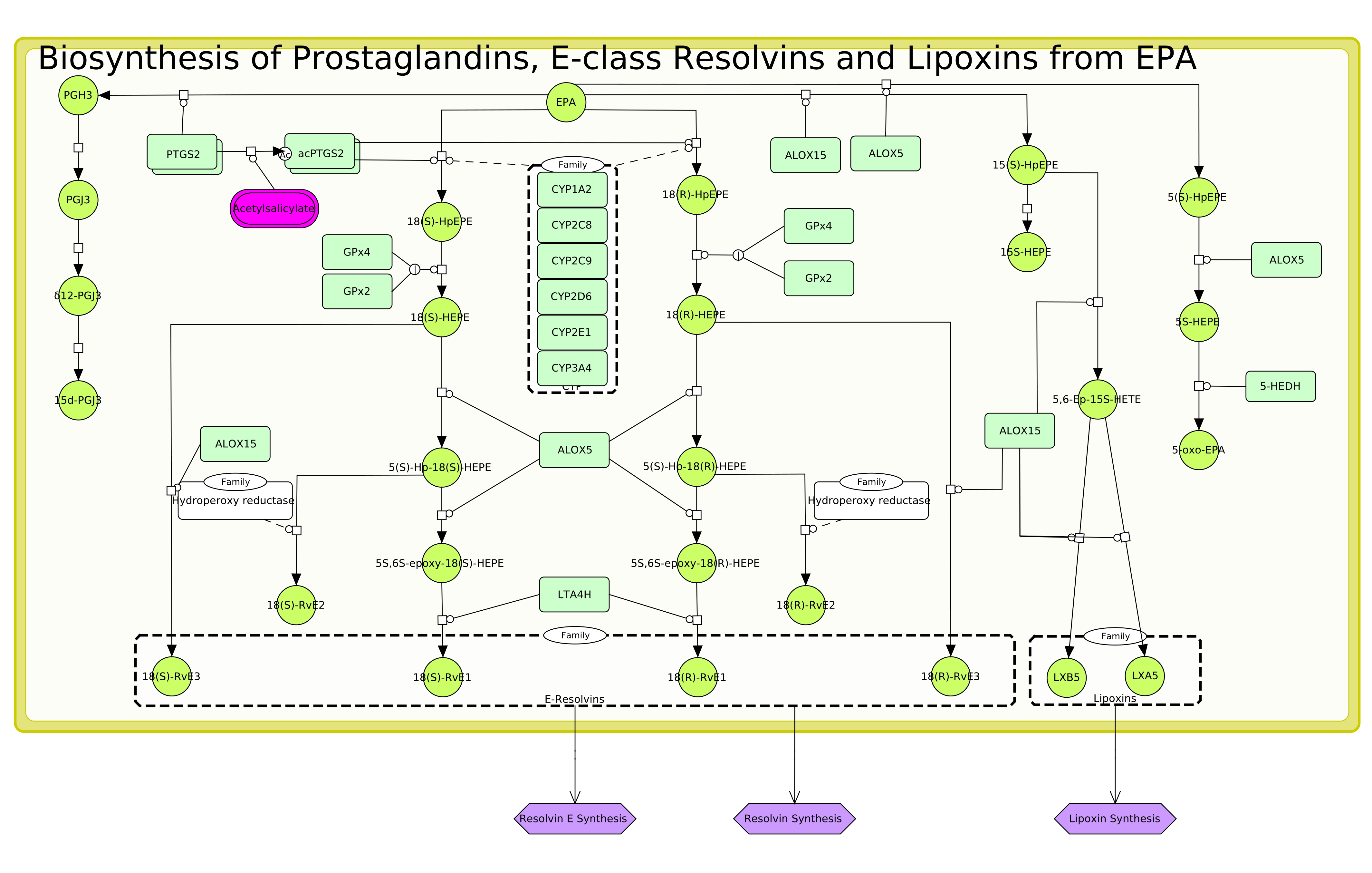

Supplement: Supplementary file 1 [file ijms-24-04342-s001.zip › FigureS3.jpg]
